# Bulk Schottky Junctions‐Based Flexible Triboelectric Nanogenerators to Power Backscatter Communications in Green 6G Networks

**DOI:** 10.1002/advs.202305829

**Published:** 2023-12-01

**Authors:** Yilin He, Amus Chee Yuen Goay, Anthony Chun Yin Yuen, Deepak Mishra, Yang Zhou, Teng Lu, Danyang Wang, Yun Liu, Cyrille Boyer, Chun H. Wang, Jin Zhang

**Affiliations:** ^1^ School of Mechanical and Manufacturing Engineering University of New South Wales Building J17, Kensington Sydney NSW 2052 Australia; ^2^ School of Electrical Engineering and Telecommunications University of New South Wales 330 Anzac Parade, Kensington Sydney NSW 2033 Australia; ^3^ Department of Building Environment and Energy Engineering The Hong Kong Polytechnic University Hung Hom Kowloon Hong Kong SAR 000 China; ^4^ Research School of Chemistry Australian National University College of Science Building 137, Sullivans Creek Rd Acton ACT 2601 Australia; ^5^ School of Materials Science and Engineering University of New South Wales Hilmer Building, Kensington Sydney NSW 2052 Australia; ^6^ School of Chemical Engineering University of New South Wales Building E8, Kensington Sydney NSW 2052 Australia

**Keywords:** 6G wireless networks, backscatter communications, energy harvesting, Schottky junction, triboelectric nanogenerators

## Abstract

This work introduces a novel method to construct Schottky junctions to boost the output performance of triboelectric nanogenerators (TENGs). Perovskite barium zirconium titanate (BZT) core/metal silver shell nanoparticles are synthesized to be embedded into electrospun polyvinylidene fluoride‐*co*‐hexafluoropropylene (PVDF‐HFP) nanofibers before they are used as tribo‐negative layers. The output power of TENGs with composite fiber mat exhibited >600% increase compared to that with neat polymer fiber mat. The best TENG achieved 1339 V in open‐circuit voltage, 40 µA in short‐circuit current and 47.9 W m^−2^ in power density. The Schottky junctions increased charge carrier density in tribo‐layers, ensuring a high charge transfer rate while keeping the content of conductive fillers low, thus avoiding charge loss and improving performance. These TENGs are utilized to power radio frequency identification (RFID) tags for backscatter communication (BackCom) systems, enabling ultra‐massive connectivity in the 6G wireless networks and reducing information communications technology systems’ carbon footprint. Specifically, TENGs are used to provide an additional energy source to the passive tags. Results show that TENGs can boost power for BackCom and increase the communication range by 386%. This timely contribution offers a novel route for sustainable 6G applications by exploiting the expanded communication range of BackCom tags.

## Graphical abstract

Triboelectric nanogenerators (TENGs) utilizing bulk Schottky junctions are developed to enhance electrical performance, with the inclusion of perovskite/silver core shell nanoparticles into PVDF‐HFP fibers. When integrated with a backscatter tag, the TENG enhances the maximum read range by a five‐fold increase, which shows proof of concept in deploying green TENG‐powered backscattering systems for 6G networks.

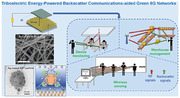

## 1 Introduction

Backscatter communication (BackCom) systems are one of the emerging technologies that enable the development of ultra‐massive connectivity from the 6th generation (6G) wireless communications. BackCom enables communication with battery‐less devices called tags by using existing radio frequency (RF) signals such as radio, TV, and mobile phones to transmit data. This utilization of ambient wireless signals for enabling BackCom enhances not only the overall spectral efficiency of the system but also supports integrated sensing and communication (ISAC) when tags are deployed for sensing applications.^[^ ^1^ ^]^ However, the short communication range strongly limits their applications due to power attenuation along a long distance. Triboelectric nanogenerators (TENGs) harness mechanical energy (e.g., human movement) and convert it to electrical outputs utilizing the triboelectric effect and become an attractive solution to recycle wasted mechanical energies during daily life to power electronics for physiological monitoring, motion tracking and human‐machine interactions with broad applications in health care, security and entertainment sectors.^[^ ^2^ ^]^ In this case, to incorporate TENG with the BackCom system is a potential renewable approach to provide the energy required by the passive backscatter tag to activate the microcontroller to modulate the backscatter signals and extend the communication range. This work therefore provides a green solution to support BackCom systems by either expanding the communication range for a given transmit power at the reader or reducing the carbon footprint by reducing the emissions from the reader for a given BackCom range.

The abundance of wasted mechanical energy in the IoT environments provides ample opportunities for energy harvesting with TENGs. Additionally, TENGs' low‐cost, simple structure, a broad choice of materials and high energy harvest efficiency to harvest low frequency and irregular mechanical energy make them suitable for various scenarios.^[^ ^3^ ^]^ Depending on the materials and structures, TENGs can be either flexible^[^ ^4^ ^]^ or rigid^[^ ^5^ ^]^; they can be used alone^[^ ^6^ ^]^ or connected in arrays to power other devices^[^ ^7^ ^]^ or as self‐driving sensors.^[^ ^8^ ^]^ By connecting a rectifier circuit, the TENGs can provide a direct current power source suitable for low‐power IoT devices, making them an ideal solution for powering energy‐efficient applications such as BackCom systems.^[^ ^2^, ^9^ ^]^ This is highly relevant and timely because, with advancements in semiconductor technology, BackCom tags are gaining many interests in indoor settings for short‐range communications where TENGs can be used to scavenge energy from underlying movements and activities. This opens up many new applications for 6G wireless networking by enabling sustainable integrated sensing and communication while maximizing spectrum and energy efficiencies. However, here it may be noted that although the TENG output performance has experienced an ongoing improvement through material design, optimization of device structure and power management strategies, the output power of TENGs is generally low due to high output impedance and low charge transfer.^[^ ^10^ ^]^ Various methods have been employed to improve the electrical performance of TENGs. For a typical contact‐separation module TENG, when its distance (*x*) between the dielectric layer and the movable electrode is small, the open‐circuit voltage *V*_OC_ and short‐circuit current *I*_SC_ can be represented by the following equations:

(1) $$V_{oc}=\frac{\sigma x}{\varepsilon }$$

(2) $$I_{sc}=\frac{S\sigma x}{d+x}$$

where σ is the surface charge density, ε is the dielectric layer's permittivity, *S* is the contact area, and *d* is the dielectric layer's thickness.^[^ ^11^ ^]^ To increase the contact area *S*, either physical or chemical methods have been employed to modify the surface morphology of the triboelectric layer by creating micro/nano features.^[^ ^12^ ^]^ For instance, Chen et al. used silicon template to give the polydimethylsiloxane (PDMS) tribo‐layer pyramidal surface morphology, which deforms the friction materials and increases the surface area when pressed.^[^ ^13^ ^]^ To generate modified PDMS films with micropillar arrays, Cheng et al. applied etching using Ar plasma, which resulted in 70% increase in output voltage.^[^ ^14^ ^]^ To increase surface charge density σ, a common method is to use tribomaterials with a significant difference in surface potential.^[^ ^15^ ^]^ Other enhancement approaches have also been actively explored, e.g., Huang et al. demonstrated that the potential generated by piezoelectric materials could affect the triboelectric charge density and lead to a higher tribo‐output.^[^ ^16^ ^]^ As a device with a capacitive structure, improving TENG's dielectric properties also helps improving its power generation capacity.^[^ ^17^ ^]^ Studies have shown that triboelectric charges are not only stored on the tribomaterial's surface, they can also enter the material's interior under the action of charge drift caused by the potential gradient.^[^ ^18^ ^]^ As long as the charge loss caused by the enhanced conductivity can be controlled, increasing the material's conductivity can increase the triboelectric layer's charge density and output. There are numerous approaches and here only named a few.^[^ ^19^ ^]^

The Schottky junction has been broadly utilized to achieve the rectifying behavior of the electrical contact attributed from directional charge movement driven by the spontaneously formed built‐in electric field at the interface^[^ ^20^ ^]^ and an increase in current output. The triboelectric charge density can be improved by the space charge polarization of perovskites by Schottky junctions.^[^ ^21^ ^]^ Our recent work on silver/semiconducting perovskite Schottky junction based TENGs (by mixing silver nanowires and Mn‐doped 0.94(Bi_0.5_Na_0.5_)TiO_3_‐0.06BaTiO_3_ nanocrystals within the polymer fiber matrix) have shown a 386% increase in output power compared with those based on neat polymer (polyvinylidene fluoride‐*co*‐hexafluoropropylene, i.e., PVDF‐HFP) fiber mat.^[^ ^22^ ^]^ It has been reported that the contact between metal (e.g., Au) and barium zirconium titanate (BZT) thin film can construct a Schottky junction and the depletion layer for the junction can be tuned by the charging or discharging of electrons at oxygen vacancies.^[^ ^23^ ^]^

PVDF‐HFP has emerged as potential tribo‐materials for TENGs due to its robust chemical resistance, biocompatibility, mechanical adaptability, and significant tribo‐negativity from the fluorine groups.^[^ ^24^ ^]^ Such polymer is amenable to electrospinning, creating flexible fibrous mats that exhibit pronounced surface roughness and increased porosity, enhancing wearability and ventilation.^[^ ^25^ ^]^ The linear extension of the polymer during electrospinning encourages the development of the preferentially oriented β phase PVDF,^[^ ^26^ ^]^ which has the optimal spontaneous polarization, thus boosting surface charge concentration.^[^ ^27^ ^]^ Among PVDF copolymers, PVDF‐HFP stands out with its superior piezoelectric coefficient (|*d*_31_/*d*_33_| >1) and elevated electromechanical interaction factor (*k*_31_ = 0.187).^[^ ^28^ ^]^

In the current study, hydrothermal reaction has been used to directly coat Ag on the surface of BZT perovskite nanoparticles. The as‐prepared Ag coated BZT core particles were used as fillers in the PVDF‐HFP solution before electrospinning into composite fiber mats. Comparing with our previous study,^[^ ^22^ ^]^ surfactants were used for homogeneous dispersion of hybrid nanofillers, reducing filler agglomeration and enhancing the mechanical properties of the fiber mats in this work. The electrospun fiber mat was then used as the tribo‐negative layer to fabricate typical contact‐separation TENGs with two pieces of aluminum (Al) foil at each side. We used the wireless identification and sensing platform (WISP) tag as the backscatter tag to verify our proposed TENG energy supply. The WISP tag can be regarded as a computational RFID tag, i.e., a microcontroller powered by radio‐frequency energy from the reader that supports sensing, computing and radio communication via backscattering. Specifically, it can transmit its unique identification and sensing data from the connected sensor when interrogated by a RFID reader. In concrete terms, the WISP tag can be treated as an IoT node that leverages BackCom to wirelessly transmit data collected by its sensing module. When TENG was integrated with the WISP tag, it extended the maximum read range of the WISP tag from 0.7 m (without the TENG) to 3.4 m, which is approximately five times the original distance. The obtained gain of 6.86 dB in the BackCom range is a significant improvement that enhances the reliability and applicability of the WISP tags. It may be noted that this significant range extension via integration of TENG is applicable to any backscattering tag because the underlying additional harvested energy from TENG can overcome the fundamental limitation of BackCom in not being able to provide sufficient radio energy for activating the passive tags placed at longer distances form the reader.

## 2 Results and Discussion

### 2.1 Morphology of Nanoparticle Fillers and Electrospun Fibers

**Figure 1:**
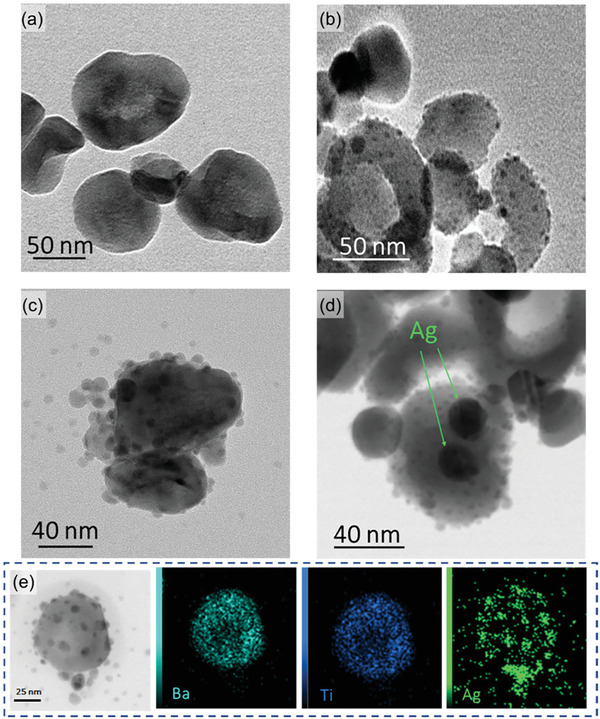
TEM images of a) pure BZT particles and BZT core/Ag coating particles b) 0.24 CS, c) 0.48 CS, and d) 0.96 CS. e) TEM image and EDS mapping of the 0.48 CS Ag coated BZT particles.

The transmission electron microscopy (TEM) images of pure BZT nanoparticles and their Ag coated particles are shown in **Figure** **1**a–d. The average size of the pure BZT particles is ≈80 nm. After reacting with 0.24 mg ml^−1^ AgNO_3_, a small amount of Ag particles appeared on the surface of the resulting 0.24 CS sample (the definition of the sample names can be found in Experimental Section of the main text). As the amount of AgNO_3_ increased to 0.48 mg ml^−1^, the number and size of Ag particles increased and scattered on the surface of BZT particles. As the AgNO_3_ concentration further increased to 0.96 mg ml^−1^, the size of Ag particles increased so that the integral Ag shell covered parts of the surface area of BZT, and the deposition of Ag particles reduced. The Energy dispersive spectrometry (EDS) mapping of a single 0.48 CS particle is shown in Figure 1e, which indicates uniform distribution of Ag particles on the surface of the BZT particle. Figure S1 (Supporting Information) shows the scanning electron microscopy (SEM) images of electrospun pure PVDF‐HFP and composite nanofibers. The comparison shows that after BZT nanoparticle addition, the fiber morphology did not vary significantly except for random protrusions, attributed from some agglomerated nanofillers. The fiber diameter was found to be 411 nm for the pure PVDF‐HFP fibers, 432 nm for the P/BZT fibers, 408 nm for the P/0.24CS fibers, 383 nm for the P/0.48CS fibers, and 323 nm for the P/0.96CS fibers. With the inclusion of conductive silver materials, the Coulomb force between the nozzle and collector and the repulsive force among the electrospun fibers increased, which contributed to finer fiber production.^[^ ^29^ ^]^ As the Ag content in the core‐shell particle increased, the above effects strengthened.

### 2.2 Electrical Outputs of TENGs

**Figure 2:**
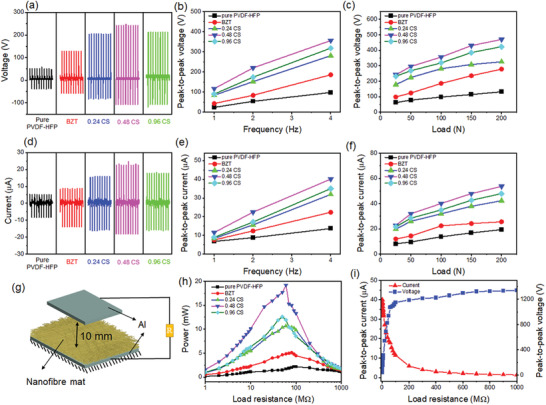
a) Voltage of TENG with PVDF‐HFP fiber mats and composite fiber mats under the impact load of 100 N and impact frequency of 4 Hz. The peak‐to‐peak voltage of TENGs with b) increasing impact frequency and c) impact load. d) Current of TENGs under impact load of 100 N and impact frequency of 4 Hz. The peak‐to‐peak current with e) increasing impact frequency and f) impact load. g) The TENG construction and the related circuit. h) The output power of TENGs versus load resistance. i) The peak‐to‐peak voltage and current of the P/0.48CS TENG with varying load resistance.

The output voltage and short circuit current data of various fiber mats tested under impact load of 100 N and impact frequency of 4 Hz are presented in **Figure** **2**a,d. The peak‐to‐peak voltage was 98 V for the pure PVDF‐HFP, 186 V for the P/BZT, 281 V for the P/0.24CS, 356 V for the P/0.48CS, and 318 V for the P/0.96CS. A similar changing trend was found for the short‐circuit current and the P/0.48CS achieved the maximum current output of 40 µA, which was 193% improvement over the TENG with pure PVDF‐HFP fiber mat as the tribo‐negative layer. Therefore, it can be inferred that the BZT particles and the BZT/Ag core‐shell particles both make a positive contribution to enhancing the TENG's output and the P/0.48CS sample showed the maximum enhancement. The charge transfer density of the P/0.48CS sample under different impact loading frequencies is shown in Figure S2 (Supporting Information), and a maximum value of 321 µC m^−2^ was reached. With the increasing Ag shell content, the output performance showed a trend of increasing at first and then decreasing. The enhanced performance was because Ag increased the dielectric properties of the TENG and resulted in a higher capacitance. At the same time, it provided fast channels for transferring surface charges to the interior of PVDF‐HFP matrix, increasing the TENG's charge density. However, as the content of Ag continued to increase, the conductive paths leading from the surface of the tribo‐layer to the bottom electrode became denser, and the chance of the charges diffusing to the bottom electrode and being neutralized by the induced charges increased significantly, so that the charge density of the triboelectric layer decreased instead, causing reduced output.^[^ ^30^ ^]^

The influences of impact frequency are shown in Figure 2b,e. With the increase in impact frequency, higher charge transfer rate was resulted in the external circuit which led to stronger current flow and improvement of the measured values of voltmeters with resistive components.^[^ ^31^ ^]^ Besides, the increased impact frequency would lower the internal impedance of the TENG and increase the circuit current and boost the voltage applied to the external devices.^[^ ^32^ ^]^ The effects of impact load on the TENGs were also investigated and the results are shown in Figure 2c,f. As the impact load increased from 25 to 200 N, all testing samples showed an increase in output voltage and short circuit current. This is because the electrospun fiber mat has inherent highly porous structure and higher pressure created more profound contacts between the tribo‐positive and tribo‐negative layer, increasing the effective contact area that improved the electrical outputs. After the impacting load exceeded 150 N, the performance enhancement slowed down, which was caused by the already compact nature of the nanofiber mats.^[^ ^24^ ^]^ Figure 2g shows the TENG construction and the testing circuit.

The output power of TENGs was measured by linking resistors (1 MΩ–1 GΩ) to the external circuit. To reduce the influence of the impedance of the test instrument on the experiment, the low‐noise current pre‐amplifier was used to measure the current, since its impedance is only 4 Ω that is negligible to the TENG itself. The tests were performed under impact load of 100 N and impact frequency of 4 Hz. The output power and voltage were calculated using the methods stated in reference^[^ ^22^ ^]^ (Figure 2h). The TENG with pure PVDF‐HFP fiber mat exhibited peak output power of 2.71 mW only with the 90 MΩ resistor. In comparison, all the TENGs with composite nanofiber mat showed significantly increased output power. In particular, the TENG with P/0.48CS layer exhibited the highest peak power of 19.17 mW with the 60 MΩ resistor, which was 600% higher than that with pure PVDF‐HFP nanofiber layer. The current and calculated open circuit voltage of the P/0.48CS TENG with varying resistance are shown in Figure 2i. The voltage curve stabilized after the load resistance exceeded 500 MΩ, and the maximum value of 1339 V at 1 GΩ was considered as the open‐circuit voltage of this TENG.

### 2.3 X‐ray Diffraction (XRD), Conductivity and Dielectric Measurements

**Figure 3:**
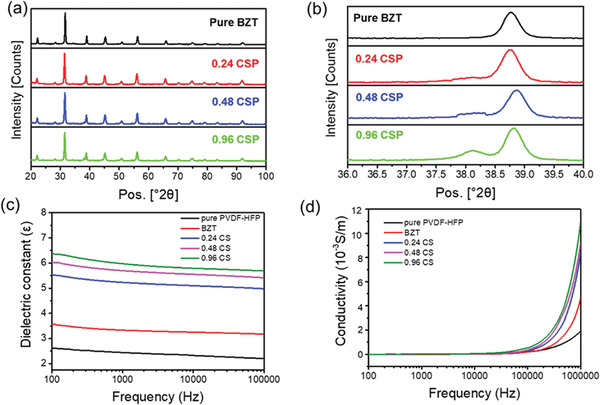
a) XRD pattern of pure BZT and Ag coated particles. b) enlarged view of XRD pattern near the Ag characteristic peak. c) The dielectric constant and d) the conductivity of neat PVDF‐HFP and their composite fiber mats.

The XRD analysis results of the pure BZT and core‐shell particles are shown in **Figure** **3**a,b. The diffraction patterns of all samples indicated the typical BZT perovskite crystalized phases. The strongest peak located at ≈2θ = 31° corresponds to the (1 1 0) plane of BZT. The minor peak at ≈45° is likely related to the cubic structure of the BZT crystals.^[^ ^33^ ^]^ The consistency of the BZT crystallographic patterns among various samples indicated that the hydrothermal treatment for the Ag coating did not affect the crystallized structure and composition of the BZT core. With the increase of Ag coating thickness, the corresponding sample's pattern near 2θ = 38° showed a gradual change from a plateau to a peak, which is related to the growing presence of the Ag (1 1 1) phase.^[^ ^34^ ^]^

As a device with a capacitive structure, the output of TENG is affected by its dielectric constant. A bigger dielectric constant leads to higher capacitance and charge density, which results in higher electrical outputs.^[^ ^35^ ^]^ Because the frequency of TENG's pulse signals produced under the contact‐separation test mode is often much higher than the frequency of the applied load, the pulse frequency is included in the frequency testing range of the dielectric constant measurements. Figure S3a (Supporting Information) shows the pulse frequency of the nanofiber mat samples under impact load of 100 N and impact frequency of 4 Hz. The results were calculated from 10 different single peaks of each sample, and the pulse frequency was 210.62 Hz for the pure PVDF‐HFP, 214.79 Hz for the P/BZT, 216.04 Hz for the P/0.24 CS, 219.16 Hz for the P/0.48CS and 215.62 Hz for the P/0.96CS. The dielectric constant curves of the fiber samples are presented in Figure 3c. With the addition of nanofillers, the dielectric constant of the fiber mats increased. Under the respective pulse signal frequency, the dielectric constant of each sample was 2.54 (pure PVDF‐HFP), 3.45 (P/BZT), 5.38 (P/0.24CS), 5.88 (P/0.48CS), and 6.22 (P/0.96CS), respectively. Compared with the neat PVDF‐HFP nanofibers, the BZT nanoparticles possess much higher dielectric constant (1000–12 000), which contributes to higher relative permittivity of their composite fiber mats.^[^ ^36^ ^]^ On the other hand, the Ag shell promotes the polarization of BZT particles during electrospinning, which further enhances the dielectric properties of the hybrid fiber mat.^[^ ^37^ ^]^ Furthermore, the creation of Schottky junctions within the composite can affect the dielectric behavior of tribo‐materials. At the interfaces where two different materials meet, charge build‐up can occur due to disparities in their respective work functions. These junctions generate a multitude of interface states capable of capturing or discharging charges. In the presence of a varying electric field, these charges can shift, leading to dielectric relaxation effects, which in turn enhance the dielectric properties. Also, the presence of Schottky barriers can instigate space charge polarization. Charges within the depletion or accumulation regions can engage with the external electric field, further boosting the material's overall polarization, and in turn, its relative permittivity.^[^ ^21^, ^38^ ^]^ All dielectric constant curves show a decreasing trend with increasing frequency, which is common in dielectric materials. As the frequency increased, the dipoles in the material had no adequate time to rotate and align in response to the electric field, which reduced the dielectric properties.^[^ ^39^ ^]^

The alternating current (AC) conductivity of nanofiber mats was characterized and recorded in Figure 3d. Within the high‐frequency range, the conductivity of the fiber mats with nanofillers significantly improved compared with that of the neat PVDF‐HFP, which may be caused by the increased electron hopping frequency and more active grain boundaries.^[^ ^37^ ^]^ In addition, as a conductive material, Ag not only enhances the conductivity of the fiber mat through tunneling (space of ≈10 nm between Ag particles) and direct contact between Ag particles but also generates more eddy currents due to rapidly changing electromagnetic fields at high frequencies.^[^ ^40^ ^]^ The enlarged view of the conductivity‐frequency curve between 100–300 Hz is shown in Figure S3b (Supporting Information). At its pulse frequency, the conductivity of each sample was 0.86 × 10^−6^ S m^−1^ (pure PVDF‐HFP), 1.56 × 10^−6^ S m^−1^ (P/BZT), 3.85 × 10^−6^ S m^−1^ (P/0.24CS), 4.51 × 10^−6^ S m^−1^ (P/0.48CS), and 5.88 × 10^−6^ S m^−1^ (P/0.96CS), respectively. The improvement of conductivity increased the TENG capacitance, which enabled more charges to be stored and enter the triboelectric layer at a faster rate, increasing the charge density of the TENG and improving the electrical output. The dielectric loss graphs of different samples are shown in Figure S4 (Supporting Information). Due to the influence of conductivity and depolarization, the dielectric loss of PVDF‐HFP increased to varying degrees after adding different fillers. Among them, the 0.96 CS sample with the highest Ag content showed the highest dielectric loss.^[^ ^41^ ^]^

### 2.4 Ultraviolet Photoelectron Spectroscopy (UPS), Charge Dynamics and Potential Measurements and Simulation

**Figure 4:**
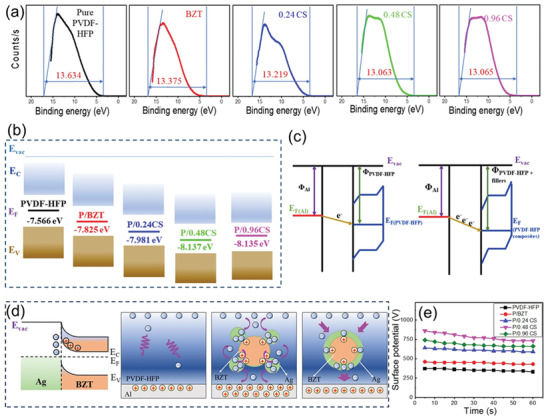
a) The UPS results of composite fiber mats. b) Schematic diagram of the energy band structure of different fiber mats. c) Illustrative graphs of energy level shifting and charge transfer at the Al‐PVDF‐HFP interface before and after filler was added. d) Comparison of electron migration between pure PVDF‐HFP and Ag coated BZT particle‐filled samples. e) Surface potential variation with time for different fiber sample after 40 s charging time.

The UPS results of different composite fiber mats are presented in **Figure** **4**a. Compared with the pure PVDF‐HFP nanofiber mat, the width of the spectrum of the fiber mats after filler addition reduced. Since the work function of materials is the difference between the incident ray energy and the binding energy, their work function enhanced accordingly, and the P/0.48CS fiber mat exhibited the highest work function of 8.137 eV. As the work function increased, the Fermi level of the material shifted closer to the valence band. When in contact with tribo‐positive materials, the band bending caused by the direct contact became more pronounced, driving more electrons from Al foil to the fiber mats (Figure 4b,c), increasing the charge density and boosting the TENGs’ performance.^[^ ^42^ ^]^ In theory, the exchange of triboelectric charges only occurs on the object's surface; however, the actual triboelectric charges are not bound on the surface of triboelectric layers, as they may drift toward the environment and into the bulk part of the material. Conductive fillers have shown to enhance the charge trapping capability, whereas excessive conductive fillers could form a continuous conductive path so the triboelectric charges can reach the bottom electrode faster through these pathways and be neutralized by the induced charges, reducing the TENGs’ output.^[^ ^43^ ^]^ By coating the BZT particles with conductive metal Ag, Schottky junctions were formed due to different free electron densities, promoting electrons to flow from the perovskite to the metal (Figure 4d).

Under the action of the electric field of the triboelectric charge and its induced charge on the bottom electrode, the space‐depletion layer of the Schottky junction near the surface of the triboelectric layer became thicker and showed a high‐resistance state, hindering the transfer of electrons from Ag to BZT. On the side close to the bottom electrode, the space‐depletion layer became thinner and showed a low‐resistance state, intensifying the transfer of electrons from BZT to Ag. This allowed the electrons to diffuse from the surface of the triboelectric layer to accumulate in Ag, which increased the carrier density of Ag, enhanced the charge transfer rate, and also increased the charge storage capacity. Besides, nanocapacitors could be created in the space between Ag and BZT. Under the electric field of the triboelectric layer and the bottom electrode, the nanocapacitors could be polarized by the internal electric field. These nanocapacitor structures were speculated to enhance the capacitance and charge density of TENGs, lowering the impedance of the TENG and increasing the current.^[^ ^21^, ^44^ ^]^ When the content of Ag was moderate, it was scattered on the surface of BZT particles, which accelerated the diffusion of surface charges to the interior and increases the charge density. As the Ag content increased further, it formed a continuous shell, which made the charges travel through these conductive paths to the bottom electrode faster, reducing the charge density.^[^ ^45^ ^]^

Because the electrostatic fieldmeter (FMX004) measures the electrical potential of an area with a depth greater than the fiber mat thickness, direct measurement of the electrical potential on the fiber mat surface causes effect of the induced charges on the bottom electrode. To eliminate the induced charges’ influence, the samples were peeled off the Al substrate and pasted on an acrylic plate for measurement. Before the potential test, the pure PVDF‐HFP samples were placed on the linear stage for charging at 4 Hz, 100 N for different times. The results are shown in Figure S5 (Supporting Information). When the charging time exceeded 30s, the surface potential tended to be stable. Therefore, the samples were prepared after charging at 4 Hz, 100N for 40s, and the surface potential measurement results are shown in Figure 4e. The surface potential of different samples increased first and then decreased with the increase of Ag content in the filler, which defends the measurement results of electrical output and work function.

**Figure 5:**
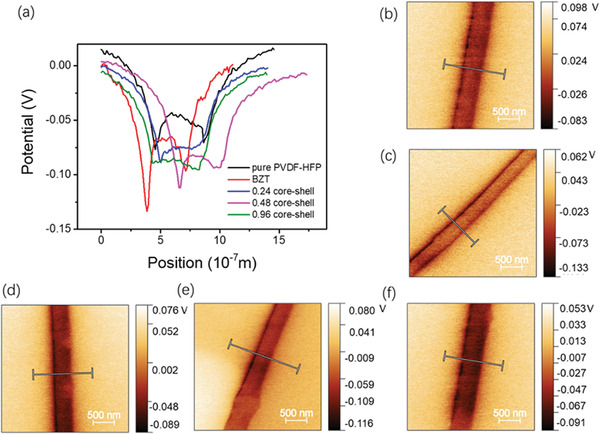
a) Surface potential distribution along the cross section direction of each single fiber. KPFM results of b) pure PVDF‐HFP, c) P/BZT, d) P/0.24CS, e) P/0.48CS, and f) P/0.96CS.

The surface potential of single fiber was measured by Kevin probe force microscopy (KPFM) and the results are presented in **Figure** **5**. As shown in Figure 5b–f, the potential on the edge of each fiber, which was caused by the changing effective area of the KPFM tip, i.e., when the probe scans cross the edge, the altitude difference between the substrate and the fiber causes the tip to tilt or even collide with the fiber.^[^ ^46^ ^]^ All fiber samples show varying degrees of edge effect depending on the sharpness of the edge and the relative probe moving direction. Figure 5a shows the potential trace of each fiber along the cross section direction. To avoid the edge influence, the highest potential value between the two major valleys was used to calculate the surface potential. In addition, due to the difference in doping ion distribution of the conductive silicon substrate, the potential of each substrate was not uniform. To reduce the errors, the fiber potential was subtracted from the substrate potential (maximum value of the potential curve), and the resulting change in potential was considered to be the surface potential of every single fiber. In this case, the potential of pure PVDF‐HFP, P/BZT, P/0.24CS, P/0.48CS, and P/0.96CS single fibers was −0.0600, −0.0652, −0.0732, −0.0856, and −0.0803 V, respectively. A lower potential of tribo‐negative materials leads to a higher potential gap from the tribo‐positive layer and boosts the TENG's performance.^[^ ^47^ ^]^ In addition, the KPFM data of pure PVDF‐HFP fiber and the fibers containing fillers at different temperatures were also compared. The results indicate that the surface potential of fiber mats was almost independent of temperature in the range of 30–80 °C (Figure S6, Supporting Information), which indicates the TENG had good thermal stability.

**Figure 6:**
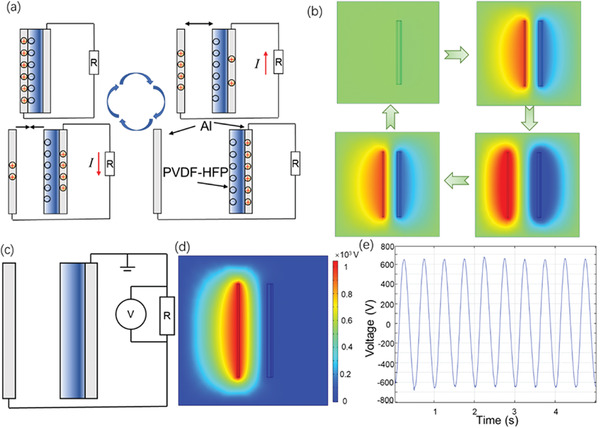
a) Schematic of the TENG's working cycle. b) Potential distribution simulation results when the tribo‐layers are at different positions. c) Simulation circuit used to measure the TENGs’ open‐circuit voltage. d) The potential distribution with the connected circuit from simulation. e) The simulated voltage curve acquired by the voltmeter in the simulation circuit.

The working mechanism of the TENG is illustrated in **Figure** **6**a, and the COMSOL simulation of the corresponding instantaneous potential distribution is shown in Figure 6b. Since PVDF‐HFP has a stronger binding force to electrons than Al foil, when the two tribo‐layers are in contact with each other, electrons on the surface of Al foil will be transferred to the surface of PVDF‐HFP while leaving an equal number of positive charges (holes) on the surface of Al foil. When the two tribo‐layers are separated from each other under the action of an external force, the binding force of the dissimilar charges to each other decreases, and two electric fields are generated in the environment. The bottom electrode in contact with PVDF‐HFP tends to retain positive charges due to electrostatic induction. Driven by the electric field, the positive charges in the top Al foil are transferred to the bottom electrode through the external circuit and create a current. As the electric field generated by the tribo‐layers increases with the increase of separation distance, the charges in the positive tribo‐layer continuously flow to the bottom electrode until they are depleted to reach equilibrium. Conversely, when the two triboelectric layers approach each other, the electrostatic induction of the bottom electrode becomes weaker, and the electrostatic binding between the two triboelectric layers becomes stronger. This causes the positive charges to transfer back toward the top electrode, creating a current in the circuit that flows in the opposite direction.

The open‐circuit voltage of the TENGs was simulated by COMSOL with the circuit shown in Figure 6c. To simulate the open‐circuit voltage, a 1GΩ resistor was connected to the external circuit, and the voltage applied to the resistor was measured by the voltmeter. The potential distribution after connecting to the circuit is shown in Figure 6d. Though the triboelectric negative layer carries negative charges due to electrostatic induction, the connected bottom electrode tends to retain positive charges to balance the potential. The simulated voltage versus time curve for the P/0.48CS sample is shown in Figure 6e. The simulation comparison between the PVDF‐HFP composite samples is shown in Figure S7 and Table S1 (Supporting Information), in which the measured dielectric constant and open‐circuit voltage values of each fiber mat were employed as inputs to evaluate the charge density and potential distribution of TENGs. The P/0.48CS sample showed the highest charge density, which matches the highest open‐circuit voltage.

### 2.5 Tensile Properties, Durability and Demonstration

**Figure 7:**
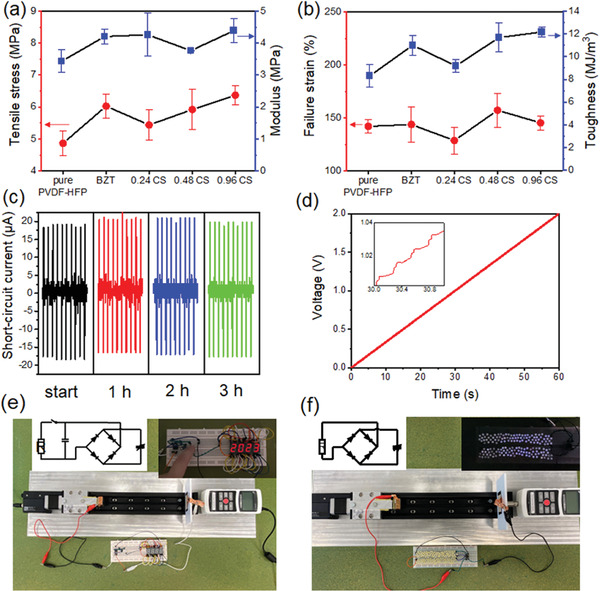
a,b) The tensile properties of nanofiber mats. c) Cyclic performance of the P/0.48CS TENG. d) Charging curve of a 35 V, 22 µF capacitor. e) The P/0.48CS TENG powers a standard seven‐segment LED display module and its circuit. f) The P/0.48CS TENG powers 160 blue LED arrays and its circuit.

The tensile properties of the fiber mats were measured since these properties are important for TENGs integrating with clothing for developing wearable electronics. Before loading the samples, the thicknesses of different fiber mats were compared using an optical microscope, and the results are shown in Figure S8 (Supporting Information). The fiber mat's thickness was considered as the average thickness of 20 sites (marked with blue lines) on the cross section. The comparison of tensile stress, Young's modulus, failure strain, and toughness between different samples is shown in **Figure** **7**a,b. All the composite fiber mats showed higher tensile stress and modulus compared to the pure PVDF‐HFP sample, which could be a result of the restricted mobility of the polymer chain with low filler concentration.^[^ ^48^ ^]^ In the case of failure strain and toughness, there was enhancement in all filler‐contained samples except the P/0.24CS sample, which may be resulted from the smallest thickness of this sample.

The cyclic testing results of the P/0.48CS TENG is presented in Figure 7c. The short‐circuit current data were collected at the start of the testing, after 1, 2, and 3 h. The impact load was 100 N and the loading frequency was 4 Hz. As the testing proceeded, there was no noticeable performance degradation, which indicates exceptional durability of the TENG made from the composite fiber mat. The comparison of the fiber surface structure before and after the cyclic testing was carried out using SEM, and the results are shown in Figure S9 (Supporting Information). It can be seen that the fiber mat after the test did not show major difference in morphology from that before the test, except that the fiber mat became denser, which explains the durable electrical performance. The first two application examples of the P/0.48CS TENG are shown in Figure 7e,f. The fiber based TENG was able to power a standard light‐emitting diode (LED) segment after charging a 35 V, 22 µF capacitor for ≈1 min. The corresponding charging curve is shown in Figure 7d, and the circuit used is shown in the inset of Figure 7e. In addition, the TENG can also directly power 160 commercial LEDs (light blue 3 mm, 15 000 millicandela). Since LEDs are unidirectional, a rectifier was used to convert the alternating current to direct current to improve power efficiency for the LED arrays.

To assess the efficacy of the TENG prepared in this work, its performance was compared with other recent TENGs that also harnessed the advantages of core‐shell fillers. This comparative analysis is shown in Figure S10 (Supporting Information),^[^ ^49^ ^]^ while the detailed information regarding the materials used in each work is cataloged in Table S3 (Supporting Information). From the data comparison, it's evident that the TENG developed in this work outperforms its counterparts and exhibits the highest output current density (Figure S10, Supporting Information).

**Figure 8:**
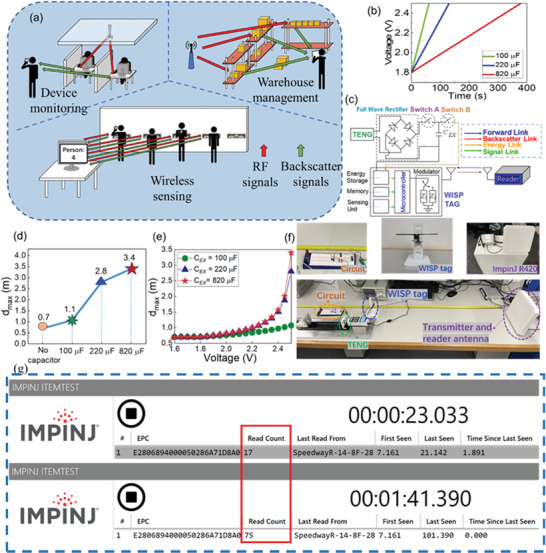
a) Illustrative diagram of several application scenarios for the backscatter communication systems. b) The voltage versus time curves for TENG charged capacitors for the next circle of powering the WISP tag. c) The charging circuit. d) Plot of the maximum read range (*d*_max_) of WISP tags without/with different capacitors. e) Effect of charged voltage on the *d*_max_ from analytical solutions. f) Read range measurement set‐up for TENG powered WISP tag. g) Comparison of the “Read Count” before and after the WISP tag activated.

The TENG was also used to power BackCom devices, which enables low‐cost passive tags to modulate the incident signal with data and reflect it back to the reader. The BackCom system typically comprises a transmitter, backscatter tags, and a reader. In the downlink phase, the transmitter broadcasts a radio frequency (RF) carrier to the backscatter tags. Upon receiving the incident RF signal, the tag modulates and reflects the signal to transmit its information to the reader in the uplink phase.^[^ ^50^ ^]^ As the tag does not require any active RF components, it can be designed to be extremely low‐cost and small in size. Moreover, passive tags that harvest energy from the incident RF carrier to support circuitry operation can further reduce costs. **Figure** **8**a illustrates some typical application scenarios of the BackCom. Specifically, electronic devices or items with an RFID tag can be monitored by the control center via an RFID reader. This information can provide details about how many devices or items are present in a particular area. In addition, RFID tags can also be utilized for warehouse inventory management, which can significantly reduce the time and effort required for stock counting. The ability to quickly and accurately identify tagged items using an RFID system eliminates the need for manual scanning of each item and helps to prevent errors in inventory tracking. Furthermore, RFID technology has evolved to a more advanced application that leverages the backscattered signal to conduct sensing. Specifically, tags can be integrated with the desired sensing modules along with adequate computing capabilities to emulate the capabilities of a full‐fledged sensor node or IoT device. Also, this integrated device will be sustainable as it can power itself from the incoming RF signals and will have relatively very low‐cost and smaller footprint as compared to a conventional IoT sensor. One such integration, which is commercially available, is called WISP and we use it in our experimental investigation.

The WISP tag operates with a minimum power consumption *P*_*L*,*min*_ = −2.96 *dBm* at its active mode, showcasing its efficient energy utilization. One notable feature of the WISP tag is its energy harvesting mechanism. It can continuously harness energy from the surrounding RF signals within a certain frequency band. The harvested energy is then stored in a capacitor, enabling the tag to accumulate and store energy over time. During the active mode, the stored energy powers the circuitry of the WISP tag after being charged to a specific voltage threshold.

To optimize energy usage, the WISP tag operates in a duty cycle, efficiently alternating between energy harvesting and backscattering stages. The duration of each cycle is primarily determined by the energy harvesting stage, which relies on the power of the incident RF signal. When the power of the RF signal is higher, the duration of each cycle becomes shorter, resulting in a higher data rate. Here, it is worth noting that the WISP tag has a critical operational threshold in terms of the minimum energy required to activate it. Therefore, given a WISP tag is solely powered by the incident RF signals from the reader, once the tag is placed beyond a certain distance from the RF signal source, i.e., the reader, where the harvested energy is lower than the minimum required energy, it will not be activated. This particular distance defines the maximum read range (denoted as *d*_max_) of the tag in the monostatic BackCom system where the RF source and reader are either the same unit or are geographically co‐located. By understanding and characterizing *d*_max_, we can determine the operational limitations of the WISP tag and optimize its placement within the BackCom system. To enhance *d*_max_of the WISP tag, we propose the integration of a TENG system with the tag for overcoming the fundamental bottleneck of limited communication range in BackCom systems. The experimental set‐up for read range measurements is shown in Figure 8f. Figure 8g shows the read count comparison before and after the WSIP tag activated.

To better support the tag, the surface area of the TENG was increased to 25 cm^2^. Furthermore, to enhance the output efficiency, the impact frequency increased to 10 Hz, while the impact load remained at 100 N. To accommodate the increased frequency, the gap between the tribo‐layers was reduced to 6 mm. Capacitors, in conjunction with a rectifier circuit, were employed to temporarily store energy generated by the TENG, enabling stable energy input for the tag, which is crucial for its optimal performance. The voltage versus time curves of various capacitors and the charging circuit are illustrated in Figure 8b,c, respectively. The circuit diagram illustrates the integration of TENG with the WISP tag. It includes two switches, allowing the TENG‐assisted WISP tag to operate in two distinct phases, i.e., the energy charging phase and the backscattering phase. During the energy charging phase, switch A is closed while switch B is opened, enabling the TENG to charge the capacitor *C*_EX_. The capacitor *C*_EX_, in conjunction with a rectifier circuit, is employed to temporarily store energy generated by the TENG before supplying power to the tag. Once the capacitor *C*_EX_ has reached a certain voltage level (2.5 V), switch A is opened, and switches B will be closed. This operation combines the harvested energy from the antenna and the energy stored in the capacitor *C*_EX_, which is then supplied to the microcontroller for signal backscattering. The modulator switches between two load impedances (*Z*_1_, *Z*_2_) to vary the current flow in the tag, thereby modulating the backscattered signal with data, known as the load modulation technique.

As shown in Figure 8d, the WISP tag exhibited a maximum read range of 0.7 m when operating without any external energy source. However, when the WISP tag was connected to capacitors with different capacitance values and charged to the voltage of 2.5 V, we observed significant extensions in *d*_max_. Specifically, the read range extended to 1.1, 2.8, and 3.4 m when connected to capacitors with capacitance values of 100, 220, and 820 µF, respectively. As the capacitance increased, the capacitor was able to store more energy at the same voltage level. Consequently, the WISP tag became less reliant on the power of the incident RF signal, resulting in an extended read range. It is important to consider the trade‐off associated with using capacitors of larger capacitance. While they offer an increased read range, they also require a longer time for the TENG to charge the capacitor to the required voltage of 2.5 V. The longer charging time leads to a longer duty cycle for the WISP tag, affecting its overall operational efficiency. Additionally, an interesting observation is that after connecting the capacitor to the WISP tag, the voltage did not discharge fully to 0 V. Instead, it stabilized at ≈1.8 V. During subsequent charging phases, the TENG therefore only needed to charge the capacitor from 1.8 to 2.5 V, resulting in shorter charging time (Figure 8b). These results demonstrate that the TENG is capable of efficiently powering the WISP tag, highlighting its potential use in BackCom wireless networks. The extension of maximum read range using TENG is critical because *d*_max_ indicates the coverage capability for the RFID system. To use inventory tracking as an example, the system with longer *d*_max_ can cover bigger area for tag reads and track more assets with fewer RFID readers. The use of TENG assisted BackCom tags will have important applications in sensing, locationing, and communication, especially when a larger backscattering range is required to minimize emissions from the reader and reduce carbon footprint. Additionally, the TENG powered BackCom systems help to reduce the number of readers or other radio and computing resources required to cover a particular area of interest. This lesser reliance on RF sources for powering the BackCom tags is in line with the recent global interests of moving toward net zero emissions and promoting the use of renewable sources of energy like TENG for enabling green sustainable communication.

Next to gain key engineering design insights, we develop new mathematical relationships between the TENG integrated tag parameters and the underlying achievable BackCom range. Specifically, analytical solutions have been proposed to visualize the relation between charged voltage of the capacitor and the *d*_max_. During the energy harvesting phase, the maximum energy that can be harvested and stored by the WISP tag is dependent on the induced voltage at its antenna. The amplitude of the induced voltage (*V*_s_) is given by:^[^ ^50^ ^]^

(3) $$V_{s}=\sqrt{8P_{a}R_{A}}$$

where *P*_a_ is the maximum allowable power that can be delivered to the tag antenna and *R*_A_ is the antenna's resistance. In general, *P*_a_ can be determined as:^[^ ^51^ ^]^

(4) $$P_{a}=SA_{e}\propto \frac{1}{d^{n}}$$

with *S* represents the power density of the electromagnetic wave at the location of the tag and *A*_e_ is the tag's antenna effective area. Specifically, *P*_a_ is inversely related to the transmission distance *d* raised to the path loss exponent of *n* (*n* = 2 for free space transmission). This relationship highlights the impact of distance on the power received by the tag in the system. In our work, we assume the free space transmission and express the harvested energy (*E*_H_) at the WISP tag as:^[^ ^52^ ^]^

(5) $$E_{H}=\frac{\alpha }{d^{2}}\propto V_{s}$$

In this context, *E*_H_ exhibits an exponential decrease as the read range increases. For the TENG‐assisted WISP tag, the total energy supplied to the WISP tag (*E*_T_) is given as:

(6) $$E_{T}=E_{H}+E_{EX}$$

where *E*_EX_ is the energy provided by the capacitor *C*_EX_, that is charged by the TENG. Knowing the minimum supply voltage to the WISP tag is 1.8 V, *E*_EX_ can be expressed as:

(7) $$E_{EX}=\left\{\begin{array}{cc} \frac{1}{2}C_{EX}\left(V^{2}-1.8^{2}\right), & if\;V\geq 1.8\\ 0, & otherwise \end{array}\right.$$

where *V* is the highest voltage of the capacitor *C*_EX_ after being charged by the TENG. Concretely speaking, the WISP tag will require a minimum total energy *E*_T,min_ to fully operate. *E*_T,min_ can be expressed as:

(8) $$E_{T,min}=\frac{\alpha }{d_{max}^{2}}+E_{EX}$$

Notably, it is clear that *d_max_* increases with *E_EX_*. To determine the improvement of WISP tag in terms of *d_max_* with the assistance of TENG, the effect of *V* on *d*_max_ for different capacitance values of *C*_EX_ (100, 220, and 820 µF) was studied. Remarkably, this investigation was carried out with actual experimental data. First, *E*_T,min_ was determined by finding *d*_max_ of WISP without any external energy provided. In our experiment, the WISP tag itself achieved *d*_max_ of 0.7 m, and hence:

(9) $$E_{T,min}=\frac{\alpha }{d_{max}^{2}}=\frac{\alpha }{0.7^{2}}$$

By substituting Equation 7 and Equation 9 into Equation 8, we can obtain:

(10) $$\frac{\alpha }{0.7^{2}}=\frac{\alpha }{d_{max}^{2}}+\frac{1}{2}C_{EX}\left(V^{2}-1.8^{2}\right)$$

Which can also be presented as:

(11) $$\alpha =\frac{C_{EX}\left(V^{2}-1.8^{2}\right)}{2\left(\frac{1}{0.7^{2}}-\frac{1}{d_{max}^{2}}\right)}$$

Next, the parameter α for each different *C*_EX_ and *d*_max_ was determined from experimental data, which is presented in Table S2. Lastly, the following expression can be obtained by rearranging Equation 11:

(12) $$d_{max}=\sqrt{{\left(\frac{1}{0.7^{2}}-\frac{1}{2\alpha }C_{EX}\left(V^{2}-1.8^{2}\right)\right)}^{-1}}$$

After the α value for each different *C*_EX_ was obtained empirically, the relationship between *d*_max_ and *V* was plotted and shown in Figure 8e.

## 3 Conclusion

We have developed a novel flexible TENG using rationally designed Schottky junctions enhancement methodology by coating perovskite BZT nanoparticles with metal Ag. With the inclusion of the hybrid nanoparticles into the polymer fibers, the TENGs with electrospun fiber mat as the tribo‐negative layer exhibited superior electrical performance. The best TENG achieved 1339 V in open‐circuit voltage, 40 µA in short‐circuit current and 47.9 W m^−2^ in power density, attributed from enhanced dielectric properties, work function, and surface potential of the nanofiber mats. Schottky junctions were constructed between the BZT nanoparticles and the smaller Ag nanoparticles distributed on their surfaces, driving electrons to transport from BZT to Ag, which not only facilitated the formation of local electric fields in the PVDF‐HFP matrix when they were used as fillers, but also increased the diffusion efficiency of surface electrons into the interior of PVDF‐HFP. Both influences were beneficial for attaining higher charge density and improving performance.

In addition, the TENGs made of hybrid fiber mats showed good mechanical properties and excellent durability. When integrated with a BackCom WISP tag, the TENG enhanced the maximum read range from 0.7 to 3.4 m, which exhibited an approximately five‐fold increase. Our proof of concept is a timely development as it improves the usefulness of BackCom tags in implementing ISAC for 6G networks by enabling the deployment of green, autonomous, and spectrally efficient TENG‐powered backscattering systems.

## 4 Experimental Section

### Materials

Polyvinylpyrrolidone (PVP, 𝑀𝑤 = 40000), silver nitrate (AgNO_3_, ≥99.0%), PVDF‐HFP pellets (𝑀𝑤 = 400000) were purchased from Merck. BZT nanoparticles (Zr_0.2_BaTi_0.8_O_3_, 99.99%, 80 nm in average size, prepared by combustion method) were purchased from the US Research Nanomaterials.

### Synthesis of Ag‐Coated BZT Particles

The Ag coated BZT nanoparticles were synthesized by using the following method. First, 500 mg BZT nanoparticles were dispersed in 100 ml glycerol by using the ultrasonication probe. After the BZT suspension cooled to room temperature under magnetic stirring, different amounts (0.24, 0.48, 0.96 mg ml^−1^) of AgNO_3_ were dissolved in the BZT suspension and underwent further magnetic stirring for 2 h to facilitate Ag nucleation. Afterward, the suspension was heated to 140 °C and remained at the same temperature for 1 h to complete the growth of Ag shells before the termination of reaction by pouring the suspension into deionized (DI) water. The resulting brown‐green suspension was settled for 24 h before supernatant removal. Finally, the precipitate was washed thoroughly with DI Water and then dispersed in N, N‐Dimethylformamide (DMF). According to the concentration of AgNO_3_ used in the reaction, the resulting particles were marked as 0.24, 0.48, and 0.96 CS, respectively.

### Electrospinning of Polymer and Composite Nanofibers

Two grams PVDF‐HFP was dissolved at 60 °C in 12 ml solvent mixture of DMF and acetone (vol:vol, 1:1) with magnetic stirring for 3 h first before 40 mg PVP was added to the polymer solution as surfactant for uniform nanofiller dispersion. Hundred milligrams core‐shell particles with different Ag concentrations were then added to the prepared solution to form suspension with 5 wt.% particle concentration. As control, the solution without any nanofillers and the suspension containing only BZT particles were prepared using the same procedure. The resultant mixtures were electrospun at working voltage of 20 kV with solution flow rate of 20 µl min^−1^. The fiber collector rotated at 300 revolutions per minute and the collector/needle distance was 10 cm. The nanofiber mats were collected after 2 h of electrospinning. The resultant different types of fibers were labelled as the pure PVDF‐HFP fibers, the P/BZT (containing only BZT particles) fibers, the P/0.24CS (containing 0.24 CS core shell nanoparticles) fibers, the P/0.48CS (containing 0.48 CS core shell nanoparticles) fibers, and the P/0.96CS (containing 0.96 CS core shell nanoparticles) fibers.

### Morphological Observation and Mechanical Testing

SEM images were taken on electrospun nanofiber mats using FEI Nova NanoSEM 450. The Amplitude Modulation KPFM was used to image single fibers. TEM was employed to examine the coated hybrid nanoparticles deposited on the carbon‐coated copper grid using the 200 kV field emission JOEL FEG 2100F. The XRD patterns were obtained by the Aeris Compact XRD Diffractometer (Malvern Panalytical) with Cu source. The fiber diameter of the electrospun nanofibers was obtained from the normal distribution histogram of 100 measurements of fiber diameter from various SEM images. ESCALAB250Xi with He I (21.2 eV) source was used to conduct the UPS on each fiber mat to obtain the binding energy. For tensile property characterization, the electrospun nanofiber mats with 10 mm in width and 20 mm in gauge length were tested in a Mark‐10 ESM 303 instrument with a crosshead speed of 1.1 mm min^−1^. The tensile results were obtained from the mean value from three different samples in each group.

### Electrical Measurements

The cyclic contact‐separation movements of the TENGs were executed on a linear stage. A force gauge (Mark‐10, M5‐200) was fixed at one end of the linear stage to support the TENG and measure the impact load. A 20 mm × 20 mm aluminum (Al) block was covered with Al foil and fixed on the other end of the linear stage to serve as the moving part, positive tribo‐layer as well as one electrode. The fiber mats were cut into rectangular pieces slightly larger than the Al impact block. The Al foil substrate (for collecting fiber mats) was kept with the sample and served as the other electrode. The fiber mat sample with Al foil was pasted on an acrylic plate, which was stuck onto the force gauge with rubber tape. The maximum separation distance between the two tribo‐layers was 10 mm. The voltage and current were recorded by an Rigol DS1074Z oscilloscope through a potential differential probe and a low‐noise current pre‐amplifier (SR570, Stanford Research System with impedance of 4 Ω). The surface contact potential of single fibers was measured by AM‐KPFM, and the surface‐induced potential of fiber mats was characterized by using the Electrostatic Fieldmeter (FMX004). The surface potential of single fibers at different temperatures was characterized by using Asylum Research, Cypher ES, Oxford Instruments. The AC conductivity and the relative permittivity were calculated based on fiber mats’ resistance and capacitance, which were measured by the LCR meter (Keysight).

### Read Range Measurement of the TENG‐Assisted WISP Tag

An ImpinJ R420 reader as the transmitter and receiver, broadcasted the RF signal constantly to the WISP tag and received the backscattered signal from the tag. The WISP tag, initially positioned closely to the flat‐panel antenna, gradually moved away until the ImpinJ R420 reader no longer picked up the backscattered signal from the tag. This was marked as the maximum read range of the WISP tag. Different capacitors were charged to a voltage of 2.5 V by the TENG and were then connected to this WISP tag. These TENG powered capacitors provided an additional source of energy to the WISP tag that helped in activating it with much lower incident RF signals from the reader. By iteratively increasing the distance between the TENG‐assisted WISP tag and the antenna, the *d*_max_ of the WISP tag for different capacitors was determined.

## Conflict of Interest

The authors declare no conflict of interest.

## Supporting information

Supporting Information

## Data Availability

The data that support the findings of this study are available from the corresponding author upon reasonable request.
