# Supplementary material for: Bulk Schottky Junctions‐Based Flexible Triboelectric Nanogenerators to Power Backscatter Communications in Green 6G Networks
Source: Adv Sci (Weinh). 2023 Dec 1;11(7):2305829. doi: 10.1002/advs.202305829 (PMC10870046; doi:10.1002/advs.202305829)
Supplement: Supplementary file 1 — Supporting Information [file ADVS-11-2305829-s001.pdf]

ADVANCED SCIENCE
Open Access

Supporting Information

for *Adv. Sci.*, DOI 10.1002/advs.202305829

Bulk Schottky Junctions-Based Flexible Triboelectric Nanogenerators to Power Backscatter Communications in Green 6G Networks

Yilin He, Amus Chee Yuen Goay, Anthony Chun Yin Yuen, Deepak Mishra, Yang Zhou, Teng Lu, Danyang Wang, Yun Liu, Cyrille Boyer, Chun H. Wang* and Jin Zhang**

Supplementary Information

Bulk Schottky Junctions-Based Flexible Triboelectric Nanogenerators to Power Backscatter Communications in Green 6G Networks

Yilin He, Amus Chee Yuen Goay, Anthony Chun Yin Yuen, Deepak Mishra, Yang Zhou, Teng Lu, Danyang Wang, Yun Liu, Cyrille Boyer,* Chun-Hui Wang,* and Jin Zhang *

Y. He, Y. Zhou, C. H. Wang, J. Zhang

School of Mechanical and Manufacturing Engineering, University of New South Wales, Ainsworth Building J17, Kensington, Sydney, NSW 2052, Australia

Email: jin.zhang6@unsw.edu.au; chun.h.wang@unsw.edu.au

A. C. Y. Goay, D. Mishra
School of Electrical Engineering and Telecommunications, University of New South Wales, 330 Anzac Parade, Kensington, Sydney, NSW 2033, Australia

A. C. Y. Yuen

Department of Building Environment and Energy Engineering, The Hong Kong Polytechnic University, Hung Hom, Kowloon, Hong Kong SAR, China

T. Lu, Y. Liu

Research School of Chemistry, Australian National University, College of Science, Building 137, Sullivans Creek Rd, Acton, ACT 2601, Australia

D. Wang

School of Materials Science and Engineering, University of New South Wales, Hilmer Building, Kensington, Sydney, NSW 2052, Australia

C. Boyer

School of Chemical Engineering, University of New South Wales, Building E8, Kensington, Sydney NSW 2052, Australia

Email: cboyer@unsw.edu.au

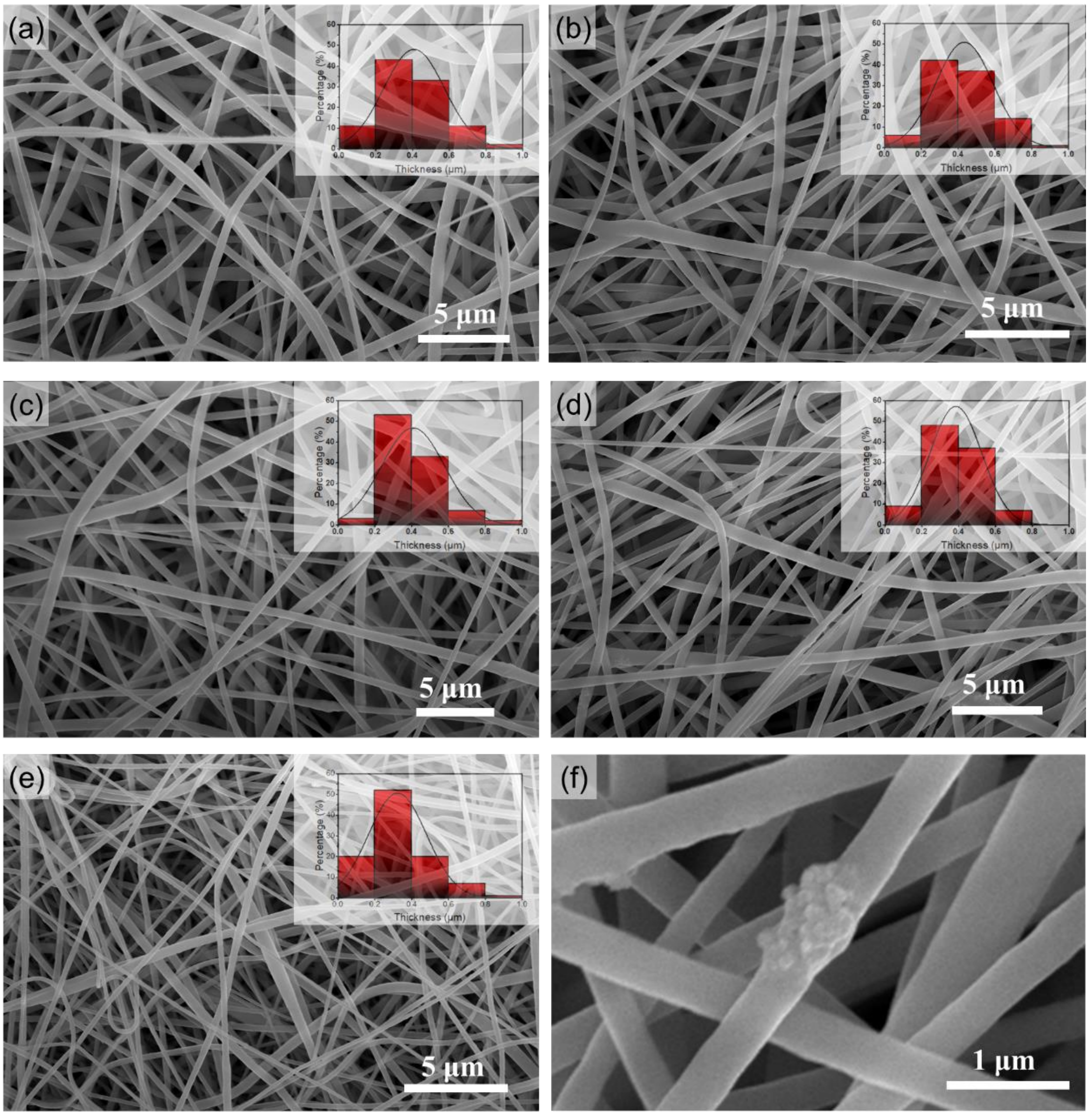

Figure S1. SEM images and fiber diameter distribution of (a) the PVDF-HFP, (b) P/BZT, (c)P/0.24 CS, (d) P/0.48CS, and (e) P/0.96CS electrospun nanofibers. The insets in a-e show the fiber diameter distribution of each type. (f) An enlarged image of the P/0.48CS sample shows agglomerated particles.

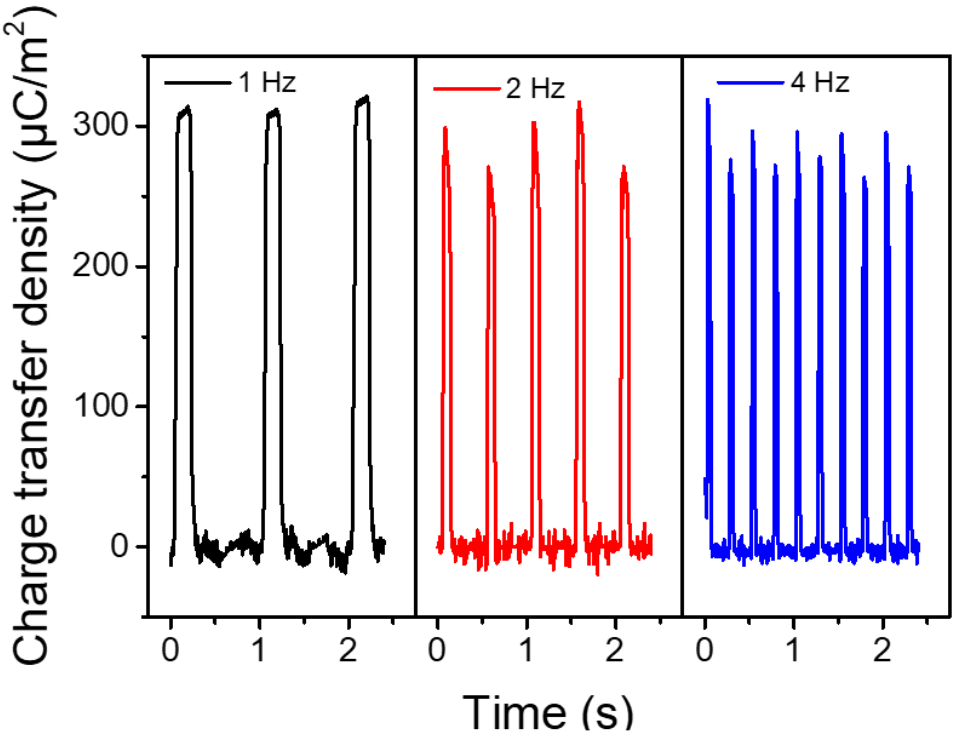

Figure S2. Charge transfer density of the P/0.48CS sample under different impact frequencies.

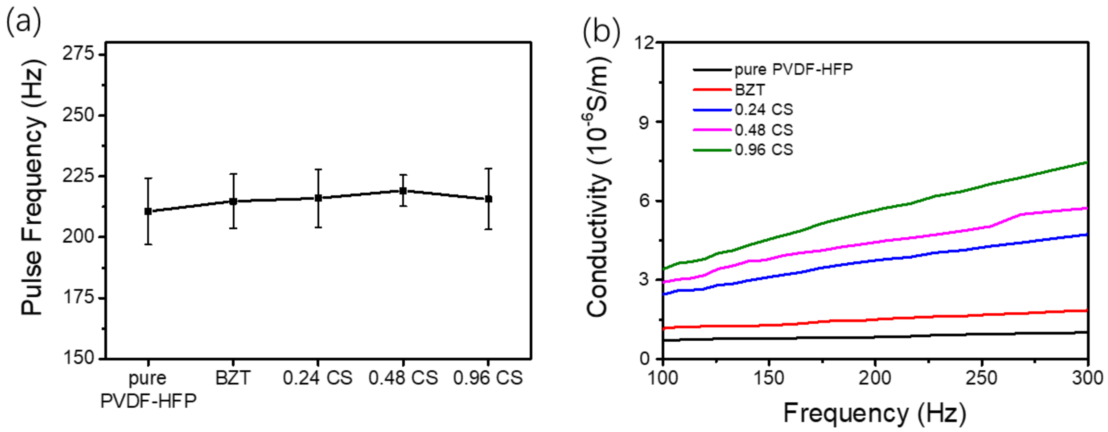

Figure S3. (a) Electrical output pulse frequency of different fiber mats under impact load of 100 N at impact frequency of 4 Hz. (b) The conductivity-frequency curve of different fiber mats in the frequency range of 100-300 Hz.

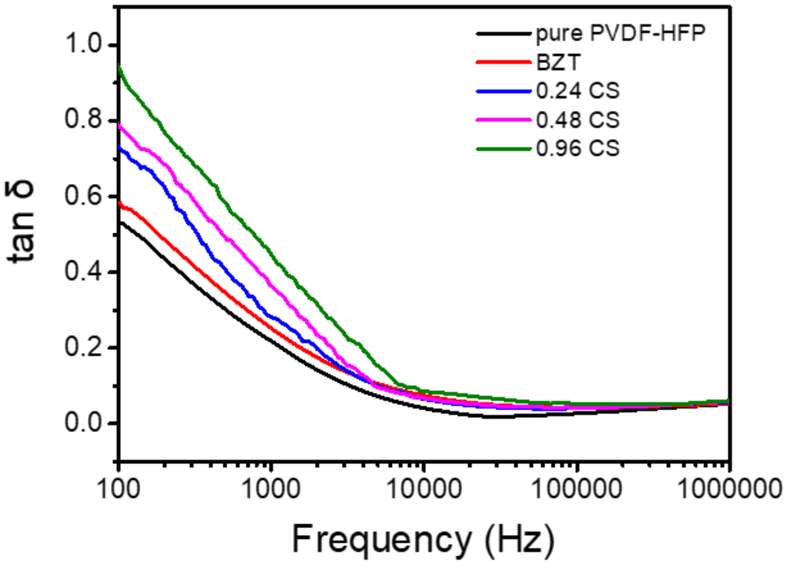

Figure S4. Dielectric loss of different fiber mat samples.

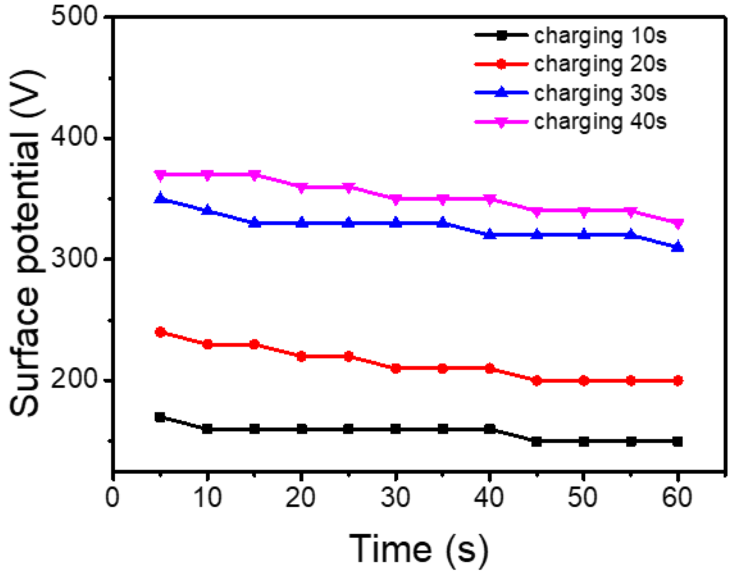

Figure S5. The surface potential variation of the pure PVDF-HFP nanofiber mat with time after different charging time.

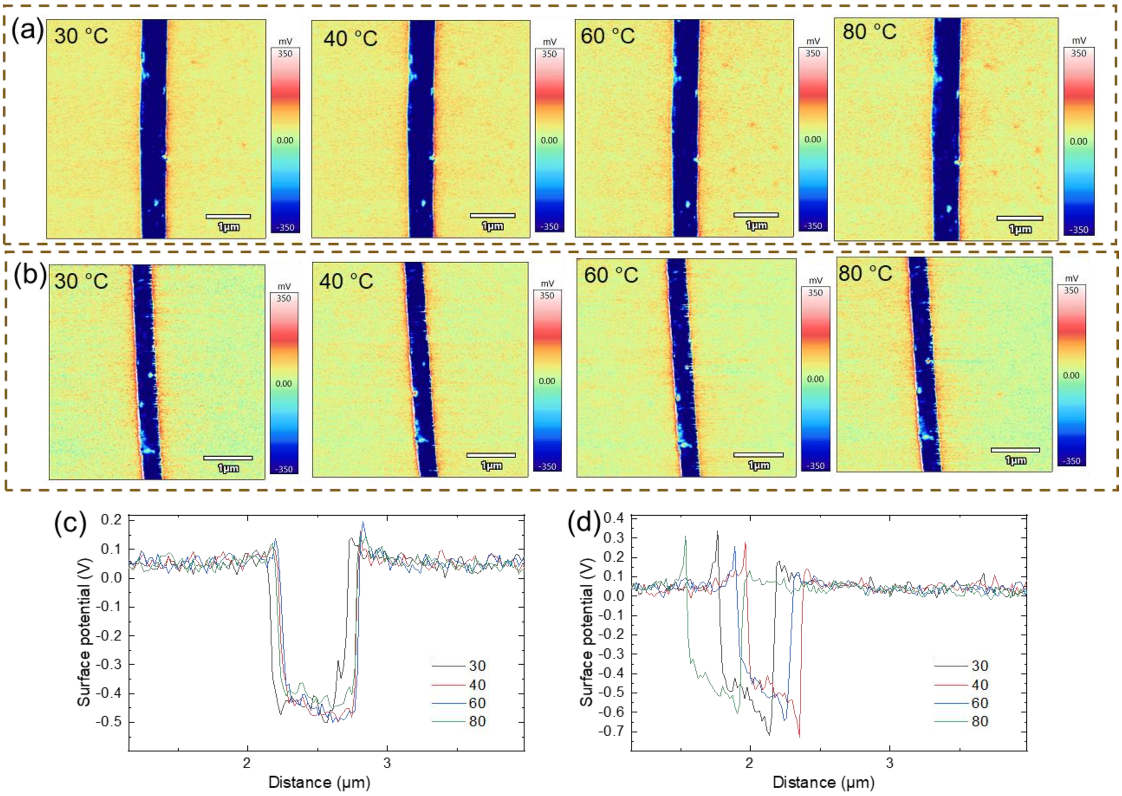

Figure S6. (a) KPFM results of pure PVDF-HFP single fiber at different temperatures. (b) KPFM results of P/0.96CS single fiber at different temperatures. (c) Surface potential distribution across a pure PVDF-HFP single fiber. (d) Surface potential distribution across a P/0.96CS single fiber.

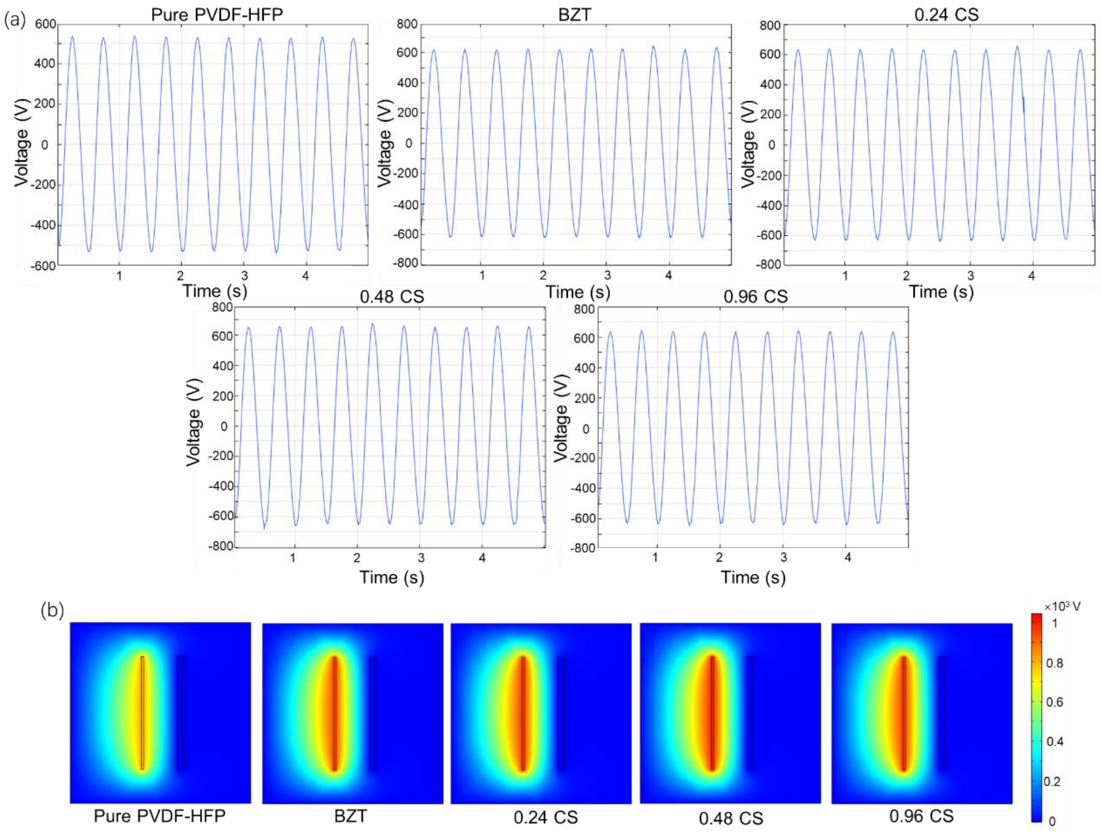

Figure S7. (a) Simulated open-circuit voltage of different fiber mat samples. (b) Potential distribution of different fiber mat samples.

Table S1. Key parameters used in the FEA simulation.

Samples	Dielectric constant	Voltage (V)	Charge density ($10^{-5}C/m^3$)
Pure PVDF-HFP	2.54	1039	3.12
P/BZT	3.45	1200	3.61
P/0.24CS	5.38	1288	3.72
P/0.48CS	5.88	1339	3.83
P/0.96CS	6.22	1307	3.73

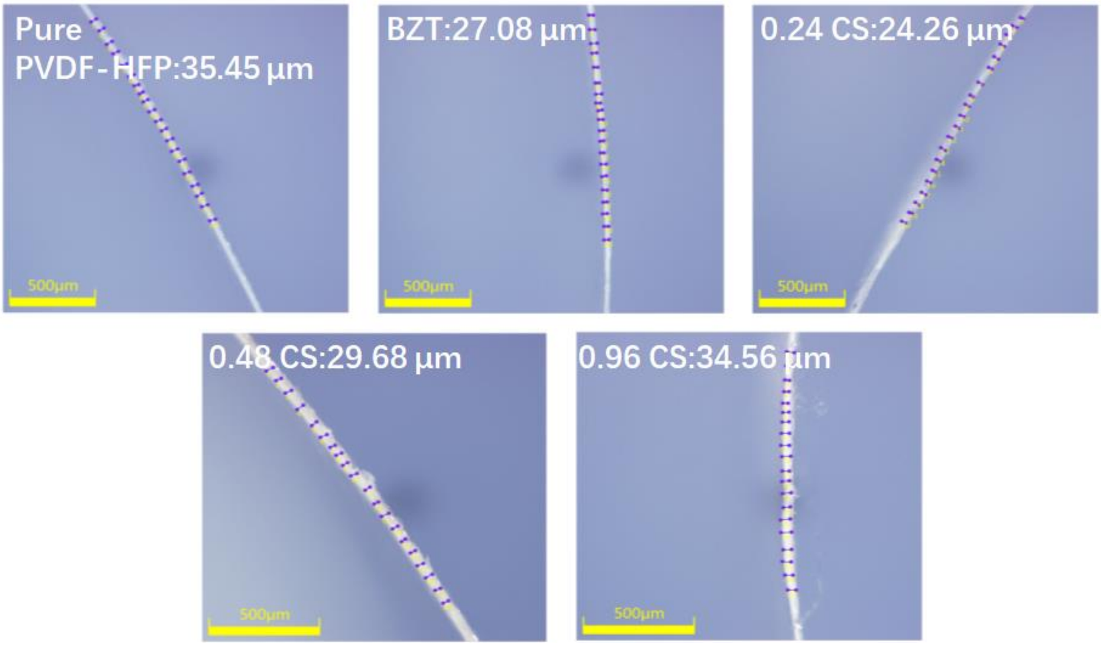

Figure S8. Optical microscope images of cross-sections of different fiber mats.

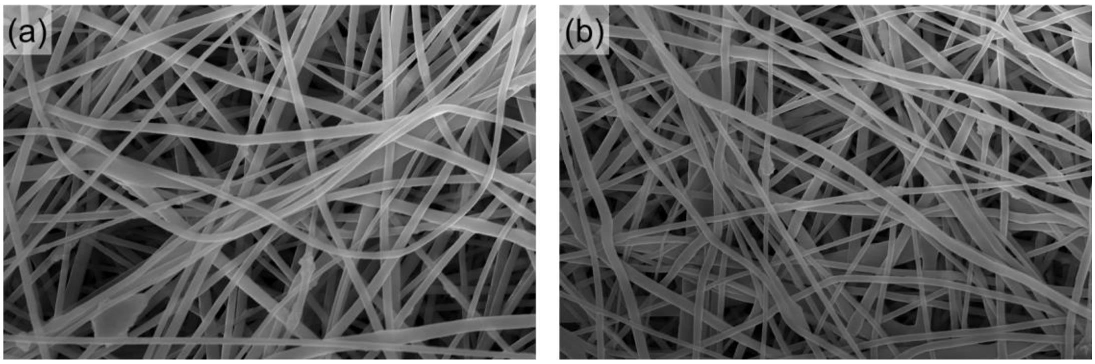

Figure S9. (a) SEM image of the P/0.48CS fiber mat sample before the contact-separation test. (b) SEM image of the P/0.48CS sample after the contact-separation tests for 3 hours.

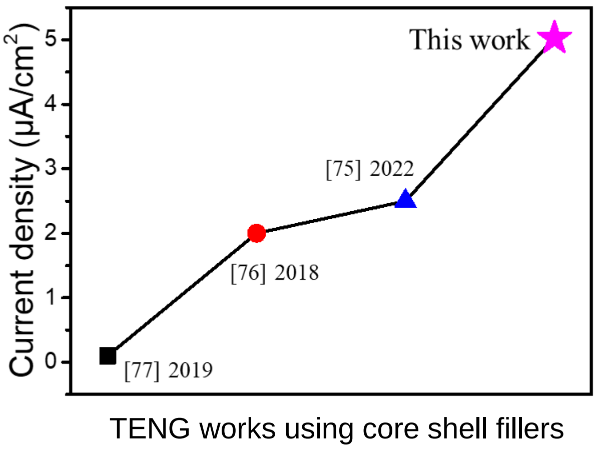

Figure S10. Current density comparison of recent core-shell filler enhanced TENGs.

Table S2. Experimental data from d_{max} measurements and the calculated α

Capacitance C_{EX} (μF)	d_{max} (m)	Value of α
Without external energy	0.7	–
100	1.07	0.1289
220	2.8	0.1731
820	3.4	0.6315

Table S3. Materials and current density of recent core-shell filler enhanced TENGs.

Matrix	Core	Shell	Output Current Density (μA/cm^2)	Reference
Polyethylene oxide (PEO)	SiO_2 microspheres	Ag	2.5	[44a]
PVDF	$BaTiO_3$ nanoparticles	Poly(tert-butyl acrylate) PtBA	2.1	[44b]
Silicone	Ga microparticles	Gallium oxide	0.1	[44c]
PVDF-HFP	BZT nanoparticles	Ag	5.025	This work
